# Methanol extract of semen *Ziziphi Spinosae* attenuates ethanol withdrawal anxiety by improving neuropeptide signaling in the central amygdala

**DOI:** 10.1186/s12906-019-2546-0

**Published:** 2019-06-24

**Authors:** Li Bo Li, Young Woo Kim, Yu Hua Wang, Li Bai, Xiao Dong Zhu, Zheng Lin Zhao, Chul Won Lee, Yu Jiao, Tong Wu, Zhen Zhen Cai, Sang Chan Kim, Won G. An, Chae Ha Yang, Guang Cheng Cui, Rong Jie Zhao

**Affiliations:** 1 Department of Psychopharmacology, School of Mental Health, Qiqihar Medical University, 333 Bukuibei Street, Jianhua District, Qiqihar, 161006 China; 2 College of Korean Medicine, Daegu Haany University, Gyeongsan, 38610 Republic of Korea; 3 Department of Pharmacology, Mudanjiang Medical University, Mudanjiang, 157011 China; 4 Department of Pharmacology, School of Korean Medicine, Pusan National University, Yangsan, 626-870 Republic of Korea

**Keywords:** Semen *Ziziphi Spinosae*, Ethanol withdrawal, Anxiety, Corticotropin-releasing factor, Nociceptin/Orphanin FQ, Amygdala

## Abstract

**Background:**

Ethanol withdrawal (EtOHW) anxiety is a crucial risk factor for alcoholic relapse. The neuropeptide nociceptin/orphanin FQ (N/OFQ) acts upon its receptor (NOP) to antagonize corticotropin-releasing factor (CRF) and elicit anxiolytic actions. Semen Ziziphi Spinosae (SZS), a prototypical hypnotic-sedative herb in Oriental medicine, exhibits anxiolytic effects during nicotine withdrawal by improving amygdaloid CRF/CRF1 receptor (CRFR1) signaling. Therefore, we evaluated the effects of SZS on EtOHW anxiety and the involvement of amygdaloid CRF/CRFR1 and N/OFQ/NOP pathways.

**Methods:**

Male Sprague Dawley rats received intraperitoneal injections of 2 g/kg EtOH (20% v/v) once daily for 28 d followed by a 3-d withdrawal. During EtOHW, the rats were given once-daily intragastric treatments of a methanol extract of SZS (MESZS, 60 or 180 mg/kg/d). Anxiety-like behaviors were measured with the open field (OF) and elevated plus maze (EPM) tests, and plasma corticosterone (CORT) levels were examined by an enzyme-linked immunosorbent assay. mRNA and protein expression levels of the neuropeptides and their receptors were determined by quantitative polymerase chain reaction and Western blot assays.

**Results:**

MESZS increased the distance traveled in the center zone of the OF and dose-dependently elongated the duration of staying in the center zone in EtOHW rats. MESZS increased both the number of entries into and the time spent in the open arms of the EPM by EtOHW rats. And, MESZS inhibited the over secretion of plasma CORT during EtOHW. EtOHW enhanced CRF and CRFR1 gene and protein expression in the central nucleus of the amygdala (CeA), which were inhibited by 180 mg/kg/d MESZS. EtOHW increased amygdaloid NOP mRNA and protein expression but spared N/OFQ mRNA expression, and 180 mg/kg/d MESZS further promoted these increases. Additionally, a post-MESZS intra-CeA infusion of either CRF or the selective NOP antagonist UFP-101 abolished the expected anxiolytic effect of 180 mg/kg/d MESZS.

**Conclusions:**

These results suggest that MESZS ameliorates EtOHW anxiety by improving both CRF/CRFR1 and N/OFQ/NOP transmissions in the CeA.

**Electronic supplementary material:**

The online version of this article (10.1186/s12906-019-2546-0) contains supplementary material, which is available to authorized users.

## Background

Alcoholism is a severe public health problem worldwide, and its treatment is greatly challenged by high rates of relapse after a prolonged period of abstinence [1]. Ethanol withdrawal (EtOHW) triggers mental disorders, such as anxiety, depression, and hyperirritability, of which anxiety in particular serves as a major negative reinforcing factor that is mainly responsible for relapse. The majority of alcoholics experience EtOHW anxiety and self-administer EtOH to alleviate the suffering [2, 3]. EtOHW rodents display substantial anxiety-like behavior in a variety of ethological tests, which precipitates EtOH seeking and self-administration [4, 5]. Hence, there is no doubt that attenuating or blocking EtOHW anxiety is an effective intervention to break the vicious abstinence–relapse cycle in alcoholics and further treat alcoholism.

The central nucleus of the amygdala (CeA) is a key limbic area for processing fear-related memory and behavior. The CeA functions as an integrative hub for anxiety [6], and the disturbed neuroendocrine transmissions in the CeA induced by chronic EtOH exposure underlie EtOHW anxiety [7]. The CeA is densely innervated by pro-stress and anti-stress neuropeptidergic systems, and imbalanced functioning of these systems during EtOHW appears to be crucial among the disturbances [8, 9]. Corticotropin-releasing factor (CRF) is the prototypical pro-stress neuropeptide, and chronic EtOH treatment abnormally elevates CRF/CRF receptor 1 (CRFR1) signaling in the CeA, which is associated with EtOHW anxiety and excessive drinking in rodents. Increased expression of both CRF and CRFR1 transcripts in the CeA of EtOHW mice [10] and intra-CeA administration of the CRF antagonist alpha-helical CRF attenuates EtOHW-induced anxiety-like behavior in rats [11]. Binge-like drinking enhances amygdaloid CRF immunoreactivity in mice [12], whereas CRFR1 antagonists block binge drinking [13]. In contrast, nociceptin/orphanin FQ (N/OFQ) is an important anti-stress neuropeptide in the CeA that produces anti-CRF and anti-anxiety actions. N/OFQ is the natural ligand for the N/OFQ peptide receptor (NOP) and is a recently discovered member of the endogenous opioid peptide family. N/OFQ and NOP exhibit different features in terms of binding affinities and biological actions, despite sharing a high degree of sequence homology with the classical opioid peptides and receptors [14]. N/OFQ and NOP produce anxiolytic and anti-EtOH dependence effects when exogenously activated. A prior intracerebroventricular injection of N/OFQ ameliorates both central CRF and EtOHW-induced increases in anxiety-like behavior in rats [15, 16]. Activation of NOP by exogenous N/OFQ inhibits reinstatement of EtOH-seeking behavior evoked by EtOH-associated cues and stress in animal EtOH self-administration models [17, 18]. Evidence indicates that functional antagonism between CRF and N/OFQ is implemented at the CeA level. For instance, prepro-N/OFQ (ppN/OFQ) and NOP mRNA levels are altered in the CeA during EtOHW, and N/OFQ inhibits the CRF-induced increase in gamma aminobutyric acid (GABA) release when iontophoretically added to ex vivo CeA slice preparations [19]. Apparently, the CRF and N/OFQ neuropeptidergic systems in the CeA constitute a promising target for treating EtOHW anxiety.

Semen Ziziphi Spinosae (SZS), the seeds of *Ziziphus jujuba* Mill. var. *spinosa* Hu ex H. F. Chou, is a prototypical tranquilizer in traditional Chinese medicine that exhibits hypnotic-sedative effects. SZS decoctions have been extensively used to treat insomnia and anxiety for over 2000 years in Oriental medicine clinical practice [20]. Instrumental analysis techniques have discovered that SZS contains a wide variety of bioactive constituents, such as C-glycoside flavones, saponins, and cyclopeptide alkaloids [21]. In laboratory animal studies, SZS crude extracts and its constituents show substantial tranquilizing effects on basal anxiety-like behavior in rodents. For example, the bioactive component Sanjoinine A in a methanol extract of SZS (MESZS) [22] increases the number of entries into and the time spent in the open arms by naive mice in the elevated plus maze (EPM) test, and the latter also increases the time spent in the central zone of the open field (OF). Moreover, pharmacological probing in these studies has demonstrated that the anxiolytic effects are ameliorated by modulating central neurotransmitters, such as GABA and 5-HT [23–25]. In addition, we have shown that an aqueous extract of SZS blocks nicotine withdrawal-induced anxiety in rats by suppressing excess activation of the amygdaloid CRF/CRFR1 pathway [26]. These findings collectively indicate that SZS treatment not only inhibits spontaneous anxiety in naive rodents, but also prevents abused drug withdrawal (stress)-induced anxiety, and the relevant mechanisms involve both neurotransmitters and neuropeptides in the brain.

Therefore, in this study, we evaluated the effect of MESZS on EtOHW anxiety and investigated the involvement of central neuropeptides with a focus on CRFergic and N/OFQergic systems in the CeA. The ultimate goal of this study was to collect and provide experimental data to develop SZS as a promising pharmaceutical to treat alcoholic anxiety and alcoholism.

## Methods

### MESZS preparation

**Fig. 1:**
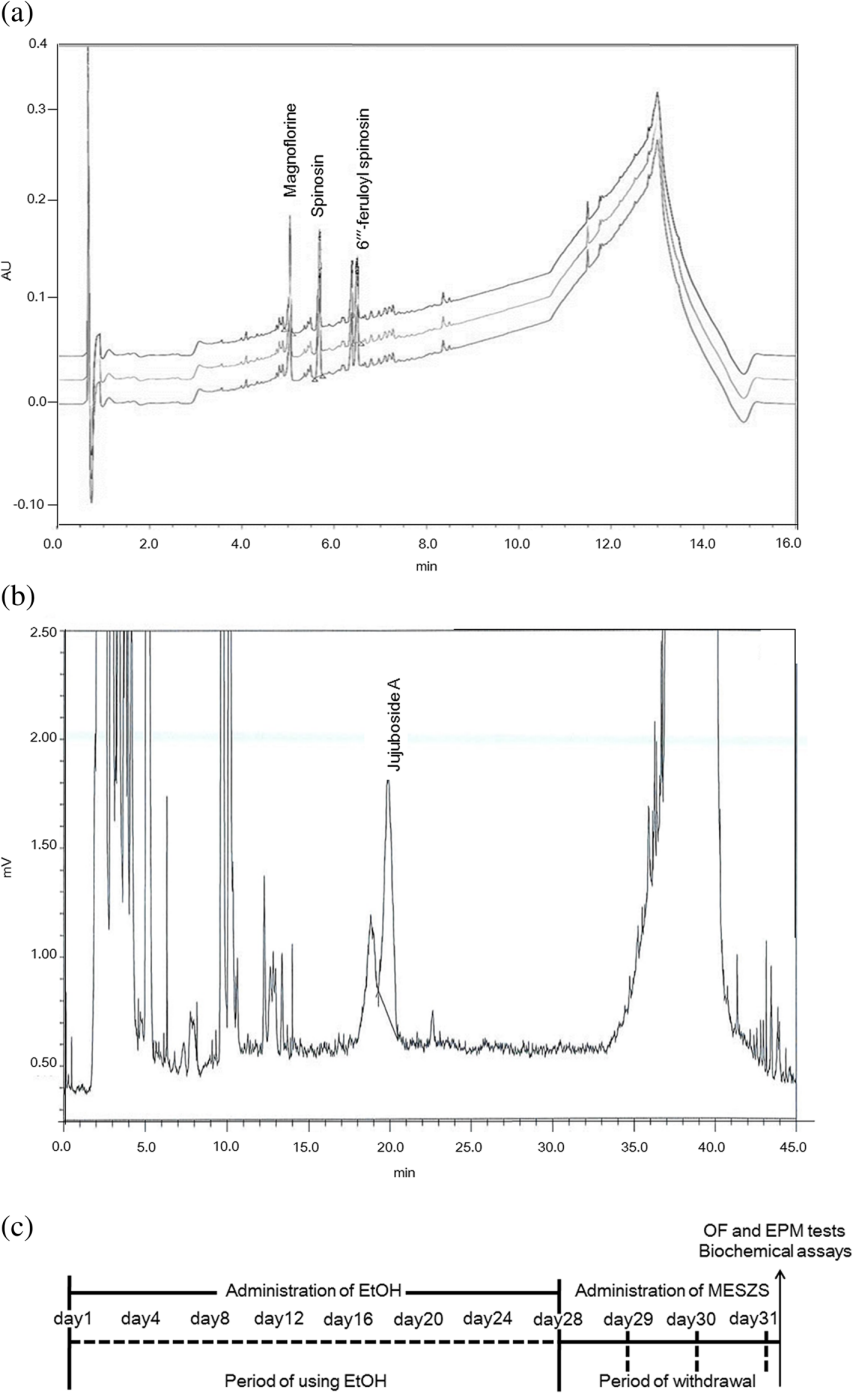
(**a**) HPLC profile of MESZS, which includes magnoflorine, spinosin, and 6′′′-feruloyl spinosin. (**b**) HPLC profile of MESZS, which includes Jujuboside A. (**c**) Time schedule for ethanol withdrawal (EtOHW). Rats were given with EtOH (2 g/kg, dissolved in saline, i.p., once a day) for 28 consecutive days followed by 3 d of withdrawal and then evaluated for behavioral and biochemical changes. During the EtOHW, the rats were administered with MESZS (60 or 180 mg/kg/d, three times). MESZS: methanol extract of Semen *Ziziphi Spinosae*; OF: open field; EPM: elevated plus maze

SZS was supplied by Daewon Pharmacy (Daegu, Republic of Korea) and its identity was verified by Professor Sang Chan Kim (Daegu Haany University; Daegu, Republic of Korea). The seeds were dry-fried, pulverized into a fine powder, immersed in 10 times the volume of methanol (1:10, w/v) for 72 h at room temperature, and further extracted using an ultrasonic cleaner for 5 h. Then, the extract was passed through filter paper (Advantec Grade No. 2 Filter Paper; Advantec, Tokyo, Japan), and was lyophilized with a vacuum evaporator. The yield of MESZS was 13.56% and analysis of the MESZS by high performance liquid chromatography (HPLC) showed that it contained spinosin, magnoflorine, 6′′′-feruloyl spinosin (Fig. 1a) and jujuboside A (Fig. 1b). A Waters Acquity Ultra Performance LC system (Waters, Milford, MA, USA) was used to analyze spinosin, magnoflorine and 6′′′-feruloyl spinosin, equipped with a Waters Acquity photodiode array detector and a Waters Acquity BEH C18 column (2.1 mm × 100 mm, 1.7 μm); the mobile phase contained acetonitrile, water and 0.1% formic acid, and the flow-rate was 0.4 mL/min. For the HPLC analysis of Jujuboside A, a Shimadzu CBM-20A HPLC System (Shimadzu, Kyoto, Japan) equipped with a Shimadzu Evaporative Light Scattering detector and an Agilent Eclipse XDB-C18 column (4.6 mm × 250 mm, 5 μm) was used, and the same acetonitrile-water mobile phase was employed and the flow-rate was 1.0 mL/min.

### Animals and experimental design

Eight-week-old male Sprague Dawley rats were obtained from the Laboratory Animal Center at Qiqihar Medical University (Qiqihar, China), and weighed 280–300 g at the beginning of the experiment. The rats were kept in a colony room (3 rats/cage) on a 12:12 h light/dark cycle with a temperature of 21–23 °C, relative humidity of 50%, and unlimited access to water and food. All experimental protocols were approved by the Animal Care and Use Committee of Qiqihar Medical University (Approval Number: QMU-AECC-2016-16) and carried out in accordance with the National Institutes of Health Guide for the Care and Use of Laboratory Animals.

EtOHW anxiety in rats often emerges as early as 6–8 h after cessation of EtOH administration, persists for several days (even longer time), and the time course of the anxiety is dependent upon the dose and duration of EtOH treatment [27]. In previous studies [28–31] and preliminary studies, EtOHW symptoms including anxiety-like behavior seemed to peak in rats 3 d after repeated exposure to intraperitoneal (i.p.) EtOH (2–3 g/kg/d for 28 d). Therefore, to broaden the observation window, in the present study, twenty-four rats received i.p. injections of EtOH (2 g/kg/d, 20% v/v, dissolved in sterile saline) for 28 d in their colony room followed by 3 d of withdrawal, and were randomly divided into three EtOH-treated groups (*n* = 8). Another eight rats were treated with sterile saline (i.p.) and assigned to the vehicle-treated control group. During the withdrawal period, the rats were orally administered either distilled water (DW) or MESZS (60 or 180 mg/kg/d, dissolved in DW) by gavage once daily for 3 d. The rats were sequentially subjected to both an OF test and an EPM test to assess anxiety-like behaviors 60 min after the final dose of MESZS (or DW). The behavioral tests were performed between 9 a.m. and 12 a.m. in a room (4.0 × 5.0 m^2^) under indirect dim light (2 × 25 W), and after the OF test, without any delay, the rat was tested in the EMP. Immediately following the EPM test, the rats were euthanized (with ether) and decapitated. Blood was collected to measure plasma corticosterone (CORT) levels and the entire brain was isolated and stored at − 80 °C until the CeA tissues were punched from the brain according to the coordinates: anterior–posterior: − 2.0 mm, medial–lateral: ± 4.2 mm, and dorsal–ventral: − 7.8 mm (based on the Paxinos and Watson Rat Brain Atlas) [32] for quantitative polymerase chain reaction (qPCR) and Western blot analyses (Fig. 1c).

### OF test

The OF test was carried out in a rectangular chamber (60 × 60 × 50 cm^3^) with dark Plexiglas walls and a mesh floor. The floor arena was geometrically divided by grid lines into nine identical squares (i.e., 20 × 20 cm^2^ zones), one center zone, and eight peripheral zones. At the start of the test, the rats were placed individually in the center zone and subsequently allowed to freely explore the whole arena for 5 min. All movements of the rats, including the total distance traveled, the distance traveled in the center zone, and the duration staying in the center zone were recorded and analyzed using a video-tracking system (Shanghai Xinruan Technology Co., Shanghai, China). The floor of each chamber was cleaned with 1% acetic acid to eliminate odor disturbances.

### EPM test

After the OF test, the rats were subjected to the EPM test as described previously [26]. The maze comprised two open arms (50 cm long × 10 cm wide, made of black acrylic) and two closed arms (enclosed by 40 cm high black walls) opposed perpendicularly by the open arms, which were elevated 50 cm above the ground and monitored with a video-tracking system (Shanghai Xinruan Technology Co.). Each rat was placed in the center of the EPM, and the numbers of arm entries and the time spent in each arm were recorded for 5 min. The percentages of the number of entries into the open arms and time spent in the open arms were calculated as follows:

$$ \mathrm{Percentage}\ {\mathrm{of}\ \mathrm{Entries}}_{\mathrm{into}\ \mathrm{open}\ \mathrm{arms}}={\mathrm{Entries}}_{\mathrm{into}\ \mathrm{open}\ \mathrm{arms}}/\left({\mathrm{Entries}}_{\mathrm{into}\ \mathrm{open}\ \mathrm{arms}}+{\mathrm{Entries}}_{\mathrm{into}\ \mathrm{closed}\ \mathrm{arms}}\right)\times 100\%, $$

$$ \mathrm{Percentage}\ {\mathrm{of}\ \mathrm{Time}}_{\mathrm{spent}\ \mathrm{in}\ \mathrm{open}\ \mathrm{arms}}={\mathrm{Time}}_{\mathrm{spent}\ \mathrm{in}\ \mathrm{open}\ \mathrm{arms}}/\left({\mathrm{Time}}_{\mathrm{spent}\ \mathrm{in}\ \mathrm{open}\ \mathrm{arms}}+{\mathrm{Time}}_{\mathrm{spent}\ \mathrm{in}\ \mathrm{closed}\ \mathrm{arms}}\right)\times 100\%. $$

### Enzyme-linked immunosorbent assay (ELISA)

First, 1 mL of blood in a chilled tube mixed with 20 μL EDTA (20 mg/mL) was centrifuged at 1500×*g* for 10 min at 4 °C. The plasma was collected and measured for CORT levels using a commercial ELISA kit (Abcam, Cambridge, UK) and the values were expressed as ng/mL.

### qPCR analysis

Total RNA was isolated from CeA tissue using Trizol reagent (Invitrogen, Carlsbad, CA, USA) and cDNA was produced from the RNA with a reverse transcription PCR kit (Promega, Madison WI, USA). Next, qPCR analysis was executed with a LightCycler® DNA Master SYBR Green-I kit (Roche Diagnostics, Mannheim, Germany) using a LightCycler 2.0 (Roche Diagnostics) according to the manufacturer’s instructions. The primers for PCR amplification of CRF, CRFR1, ppN/OFQ, and NOP were as follows: 5′-CTCTCTGGATCTCACCTTCCAC-3′ (sense) and 5′-CTAAATGCAGAATCGTTTTGGC-3′ (antisense) for CRF; 5′-GTCTCCAGG GTCGTCTTCAT-3′ (sense) and 5′-CGGACCTCACTGTTCAGAA-3′ (antisense) for CRFR1; 5′-TGCAGCACCTGAAGAGAATG-3′ (sense) and 5′-CAACTTCCGGGCTGACTTC-3′ (antisense) for ppN/OFQ; 5′-AGCTTCTGAAGAGGCTGTGT-3′ (sense) and 5′-GACCTCCCAGTATGGAGCAG-3′ (antisense) for NOP receptor; and 5′-GTCGTACCACTGGCATTGTG-3′ (sense) and 5′-GCCATCTCTTGCTCGAAGTC-3′ (antisense) for β-actin. The results were normalized to the housekeeping gene β-actin, and relative gene expression was calculated 2^−ΔΔCT^ method with the following formula:

$$ \Delta \mathrm{CT}={\mathrm{CT}}_{\mathrm{CRF}}-{\mathrm{CT}}_{\upbeta \hbox{-} \mathrm{actin}},\Delta \Delta \mathrm{CT}={\Delta \mathrm{CT}}_{\mathrm{treated}}-{\Delta \mathrm{CT}}_{\mathrm{vehicle}}. $$

### Western blot analysis

CeA tissue was homogenized in RIPA lysis buffer containing protease inhibitors and centrifuged at 16,000×*g* for 20 min at 4 °C. The total protein level in the supernatant was determined with the bicinchoninic acid assay, and the proteins were separated by 12% sodium dodecyl sulfate polyacrylamide gel electrophoresis before being transferred to polyvinylidene difluoride membranes (Millipore, Bedford, MA, USA). The membranes were first incubated with the following primary antibodies: rabbit polyclonal antibody to CRF (Abcam), rabbit polyclonal antibody to CRFR1 (Abcam), rabbit polyclonal antibody to NOP (Abcam), or rabbit polyclonal antibody to β-actin (Abcam). The membranes were incubated with horseradish peroxidase-conjugated goat anti-rabbit secondary antibody. The specific protein bands were visualized using enhanced chemiluminescence (Amersham Biosciences, Buckinghamshire, UK), and their densities were analyzed with ImageJ software (NIH, Bethesda, MD, USA).

### Intra-CeA microinfusions

To determine whether the effects of MESZS on EtOHW-induced anxiety were mediated by the amygdaloid CRF/CRFR1 and N/OFQ/NOP pathways, bilateral intra-CeA microinfusions of CRF (0.2 μg/0.2 μL on each side; Sigma Chemical Co.) or a selective NOP antagonist UFP-101 ([Nphe^1^, Arg^14^, Lys^15^] nociceptin-NH_2_) (0.1 μg/0.2 μL on each side; Tocris Bioscience, Bristol, UK) dissolved in modified Ringer’s solution (MRS; containing 150 mM NaCl, 3.0 mM KCl, 1.4 mM CaCl_2_, and 0.8 mM MgCl_2_ in 10 mM phosphate buffer, pH 7.2) were delivered 60 min after the third MESZS treatment. For this experiment, thirty male Sprague Dawley rats (280–300 g) were randomly divided into 5 groups (*n* = 6): (1) saline/DW/MRS group, (2) EtOH/DW/MRS group, (3) EtOH/180 mg/kg/d MESZS (MESZS180)/MRS group, (4) EtOH/MESZS180/CRF group, (5) EtOH/MESZS180/UFP-101 group. And, stainless steel guide cannulae (22-gauge) were bilaterally implanted into the brain with the cannula tips situated 2 mm above the CeA using a stereotaxic surgery frame under anesthesia with sodium pentobarbital (50 mg/kg, i.p.) for the intra-CeA infusions of the drugs. After the surgery, the rats were caged individually, and administered antibiotics (bacitracin ointment and penicillin) and acetaminophen for 3 d to prevent possible infection and pain. After 7 d of recovery, the rats underwent the same EtOHW (or saline) and drug treatment schedule as mentioned above (Fig. 1c). A 28-gauge injector (2 mm longer than the guide cannulae) was inserted into each guide cannula, and the drugs were introduced using a motorized syringe pump (over 60 s). The rats were tested in the EPM 5 min after drug administration, and the cannula positions of each rat were verified by histological examination for their correct location at the end of the test (see the Additional file 1: Positions of CeA cannulae).

### Statistical analysis

All data are given as the mean ± standard error of the mean. The data were statistically analyzed by one-way analysis of variance, and post-hoc comparisons were made using the Newman–Keuls multiple-comparison test using GraphPad Prism 5.0 software (GraphPad Software; San Diego, CA, USA). *P*-values < 0.05 were considered significant.

## Results

### Effects of MESZS on EtOHW-induced anxiety-like behavior

In preliminary experiments, MESZS doses > 180 mg/kg/d significantly reduced locomotor activity and compromised memory performance in rats when given once daily for 3 d (data not shown). A single dose of 180 mg/kg MESZS attenuated basal anxiety-like behavior in rats in the EPM test. Hence, in this study, 180 mg/kg/d MESZS was selected as the high dose (data not shown).

**Fig. 2:**
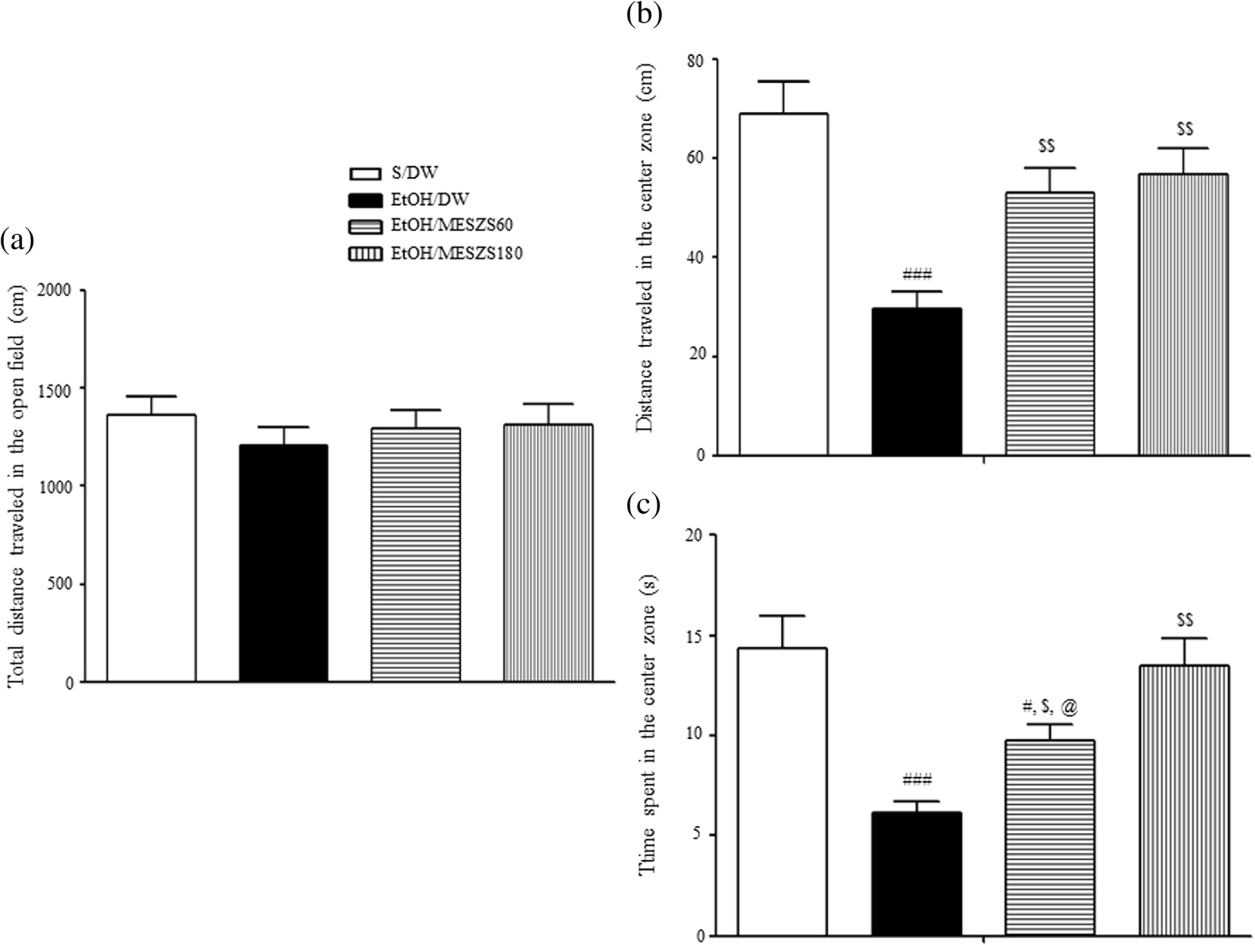
Effects of MESZS on the behavioral changes of rats in the open field test. All data are expressed as a mean ± SEM (*n* = 8). (**a**) The total distance traveled in the open field by rats. (**b**) Distance traveled in the center zone. (**c**) Time spent in the center zone. S: saline, DW: distilled water, MESZS: methanol extract of Semen *Ziziphi Spinosae*, MESZS60: 60 mg/kg/d MESZS, MESZS180: 180 mg/kg/d MESZS. ^#^ *p* < 0.05 and ^###^*p* < 0.001 vs. S/DW group; ^$^ *p* < 0.05 and ^$$^ *p* < 0.01 vs. EtOH/DW group; ^@^ *p* < 0.05 vs. EtOH/MESZS180 group (one-way ANOVA followed by Newman-Keuls post hoc test)

Rodents display a natural aversion to open areas, but they have an innate drive to explore novel environments; therefore, the degree of anxiety in the OF test is negatively associated with locomotion activity, as well as the visiting frequency and stay in the center zone. In this study, 3 d after terminating EtOH, the EtOHW rats exhibited significant decreases in the distance traveled in the center zone and the time remaining in the center zone compared with saline-treated control rats [distance traveled in the center zone: F_(3, 28)_ = 10.21, *p* < 0.001; saline-treated control group (68.88 ± 6.47, *n* = 8) vs. EtOH-treated control group (29.75 ± 3.27, *n* = 8), *p* < 0.001; time staying in the center zone: F_(3, 28)_ = 10.61, *p* < 0.001; saline-treated control group (14.38 ± 1.59, *n* = 8) vs. EtOH-treated control group (6.13 ± 0.55, *n* = 8), *p* < 0.001]. However, these anxiety indices were improved with MESZS (60 and 180 mg/kg/d) treatment, with a dose-dependent effect on the time remaining in the center zone [distance traveled in the center zone: EtOH-treated control group vs. EtOH/MESZS60 group (53.13 ± 4.96, *n* = 8), *p* < 0.01; EtOH-treated control group vs. EtOH/MESZS180 group (56.75 ± 5.27, *n* = 8), *p* < 0.01; time staying in the center zone: EtOH-treated control group vs. EtOH/MESZS60 group (9.75 ± 0.82, *n* = 8), *p* < 0.05; EtOH-treated control group vs. EtOH/MESZS180 group (13.50 ± 1.38, *n* = 8), *p* < 0.001; EtOH/MESZS60 group vs. EtOH/MESZS180 group, *p* < 0.05], indicative of therapeutic effects of MESZS against EtOHW-induced anxiety (Fig. 2). No difference was detected in the total distance traveled among the groups [total distance traveled: F_(3, 28)_ = 0.44, *p* > 0.05] (Fig. 2).

**Fig. 3:**
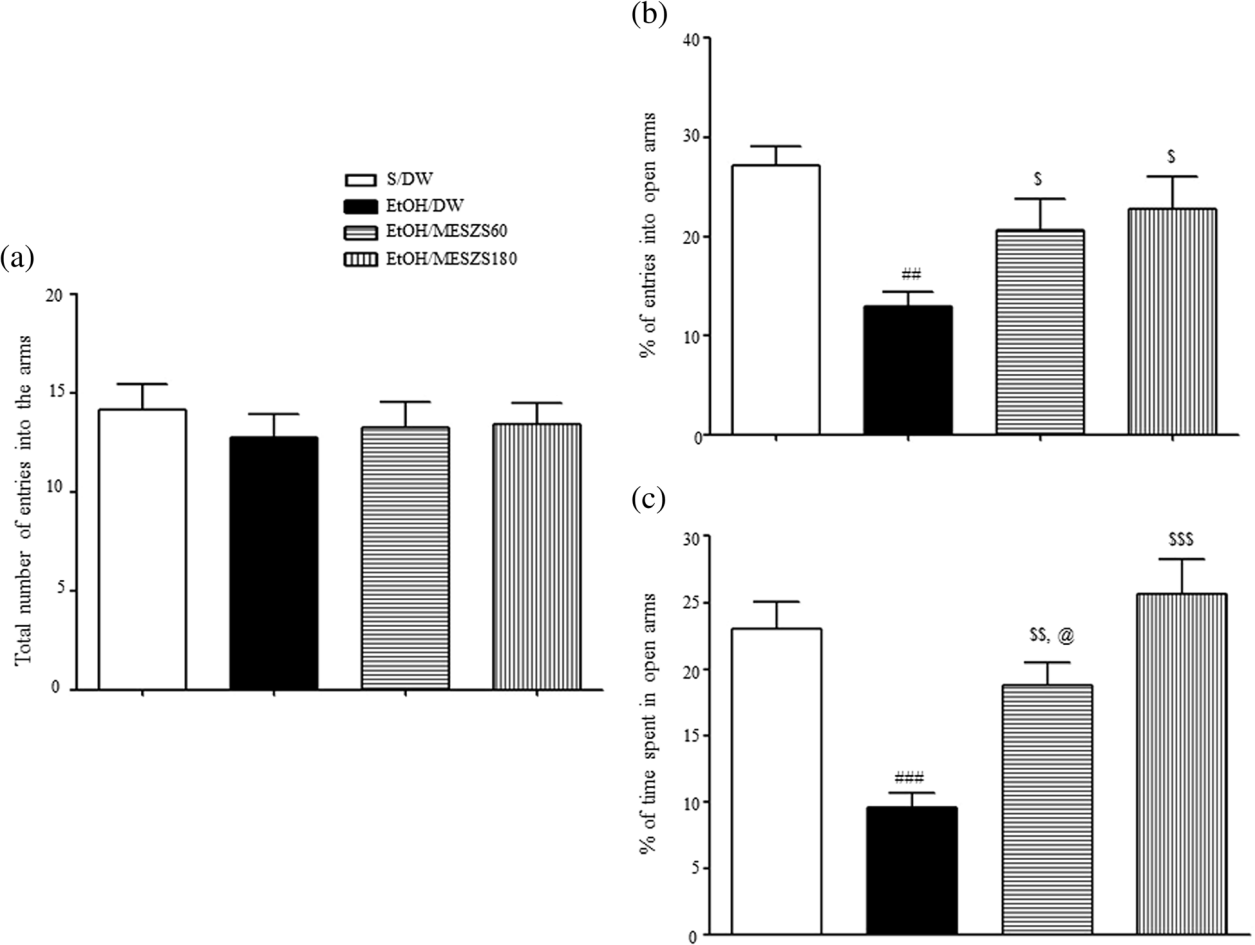
Effects of MESZS on the behavioral changes of rats in the elevated plus maze test. All data are expressed as a mean ± SEM (*n* = 8). (**a**) The total number of entries into (open and closed) arms of EPM by rats. (**b**) Percentage of numbers of entries into open arms. (**c**) Percentage of time spent in open arms. S: saline, DW: distilled water, MESZS: methanol extract of Semen *Ziziphi Spinosae*, MESZS60: 60 mg/kg/d MESZS, MESZS180: 180 mg/kg/d MESZS. ^##^ *p* < 0.01, ^###^ *p* < 0.001 vs. S/DW group; ^$^ *p* < 0.05, ^$$^ *p* < 0.01, ^$$$^ *p* < 0.001 vs. EtOH/DW group; ^@^ *p* < 0.05, vs. EtOH/MESZS180 group (one-way ANOVA followed by Newman-Keuls post hoc test)

The MESZS anxiolytic effects were corroborated by subsequent EPM tests. The EtOHW rats had fewer entries into the open arms and spent less time in the open arms than the saline-treated control rats [percentage of entries into open arms: F_(3, 28)_ = 5.39, *p* < 0.01; saline-treated control group (27.13 ± 1.95%, *n* = 8) vs. EtOH-treated control group (12.92 ± 1.54%, *n* = 8), *p* < 0.01; percentage of time spent in open arms: F_(3, 28)_ = 13.66, *p* < 0.001; saline-treated control group (23.05 ± 1.97%, *n* = 8) vs. EtOH-treated control group (9.55 ± 1.09%, *n* = 8), *p* < 0.001]. In agreement with the OF test results, both 60 and 180 mg/kg/d MESZS attenuated these anxiety-like behaviors, with a dose-dependent effect on the time spent in open arms [percentage of entries into open arms: EtOH-treated control group vs. EtOH/MESZS60 group (20.66 ± 3.09%, *n* = 8), *p* < 0.05; EtOH-treated control group vs. EtOH/MESZS180 group (22.82 ± 3.25%, *n* = 8), *p* < 0.05; percentage of time spent in open arms: EtOH-treated control group vs. EtOH/MESZS60 group (18.78 ± 1.69%, *n* = 8), *p* < 0.01; EtOH-treated control group vs. EtOH/MESZS180 group (25.66 ± 2.58%, *n* = 8), *p* < 0.001; EtOH/MESZS60 group vs. EtOH/MESZS180 group, *p* < 0.05] (Fig. 3). The total numbers of entries into the open and closed arms of the EPM by rats was not different among the groups (the total number of entries into the arms of EPM: F_(3, 28)_ = 0.23, *p* > 0.05; Fig. 3).

### Effects of MESZS on plasma CORT levels during EtOHW

**Fig. 4:**
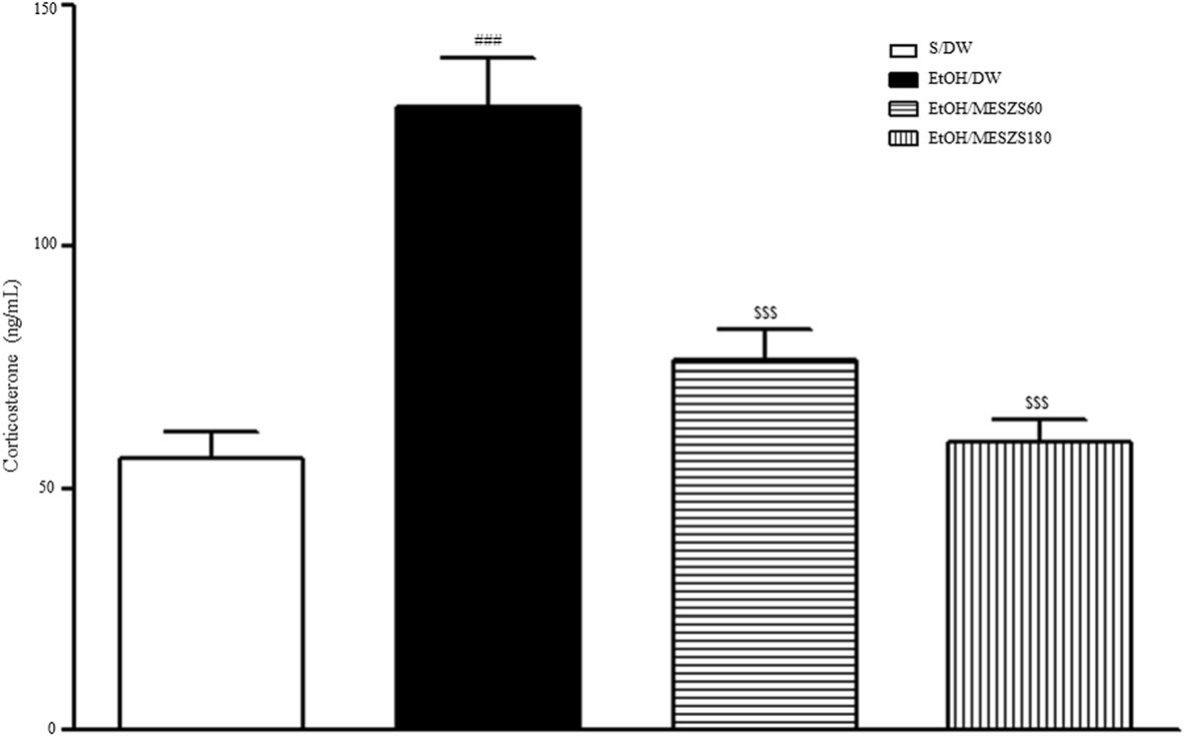
Effects of MESZS on plasma CORT concentrations during EtOHW. Withdrawal from chronic EtOH administration increased plasma CORT levels in rats, which was prevented by MESZS treatment. All data are expressed as a mean ± SEM (*n* = 8). S: saline, DW: distilled water, EtOH: ethanol, CORT: corticosterone; MESZS: methanol extract of Semen *Ziziphi Spinosae*, MESZS60: 60 mg/kg/d MESZS, MESZS180: 180 mg/kg/d MESZS. ^###^ *p* < 0.001 vs. S/DW group; ^$$$^ *p* < 0.001 vs. EtOH/DW group (one-way ANOVA followed by Newman-Keuls post hoc test)

Increased plasma CORT levels are hormonally indicative of an anxious state in rats. The ELISA results showed that the plasma CORT levels in EtOHW rats increased significantly compared to those in the saline-treated control rats [F_(3, 28)_ = 22.92, *p* < 0.001; saline-treated control group (56.04 ± 5.63, *n* = 8) vs. EtOH-treated control group (129.05 ± 10.20, *n* = 8), *p* < 0.001)], demonstrating the exacerbated anxiety behavior during EtOHW. However, the same ELISA analysis showed that these increases were inhibited by both doses of MESZS (60 and 180 mg/kg/d) [EtOH-treated control group vs. EtOH/MESZS60 group (76.36 ± 6.39, *n* = 8), *p* < 0.001; EtOH-treated control group vs. EtOH/MESZS180 group (59.50 ± 4.65, *n* = 8), *p* < 0.001], supporting the anxiolytic actions of MESZS in the behavioral tests (Fig. 4).

### Effects of MESZS on CRF and CRFR1 mRNA and protein expression levels in the CeA

**Fig. 5:**
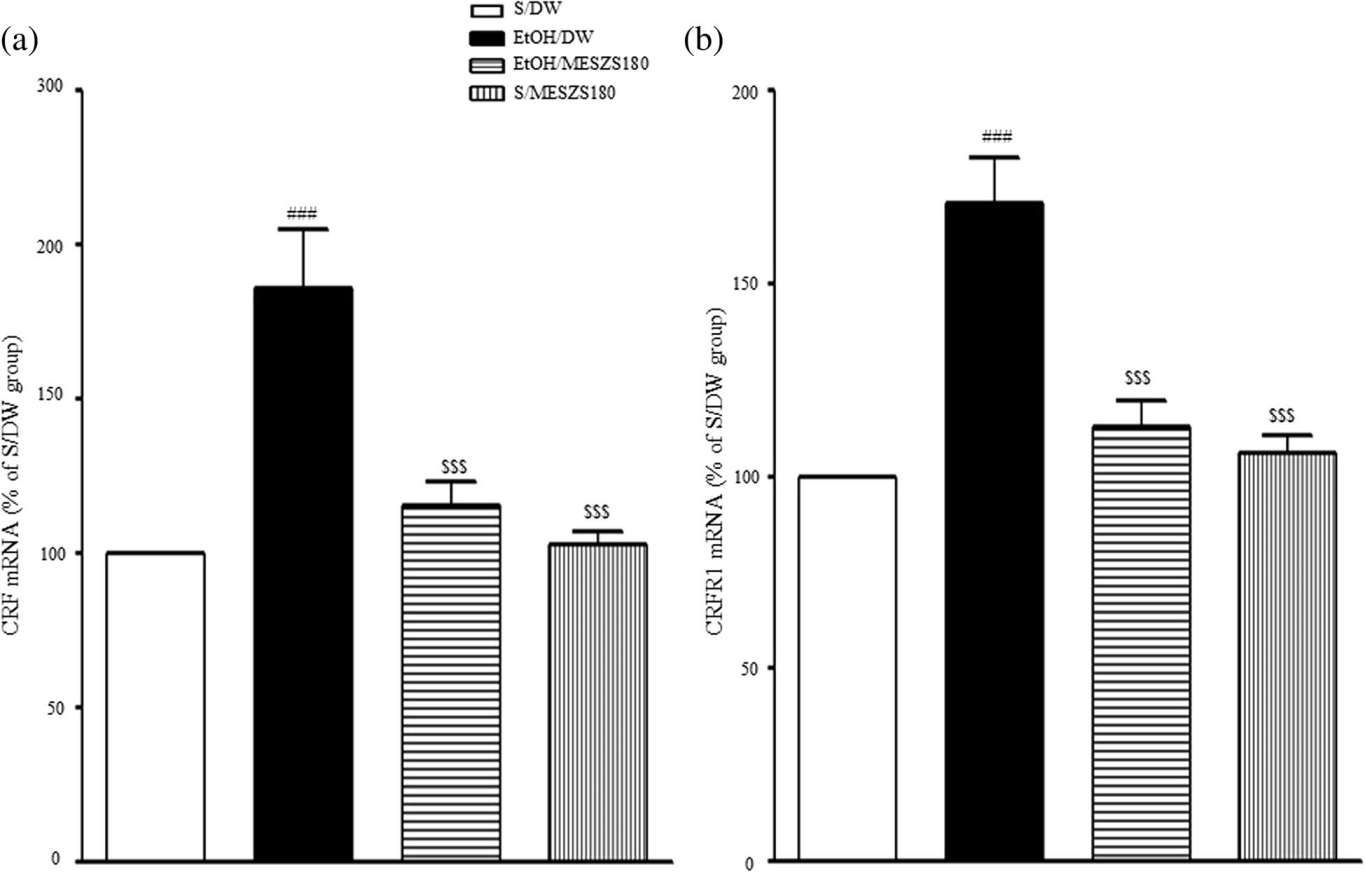
Effects of MESZS on the mRNA expressions of amygdaloid (**a**) CRF and (**b**) CRFR1 during EtOHW. Immediately after the behavioral test, the mRNA levels of CRF and CRFR1 in the CeA were determined using qPCR analysis. All data are expressed as a mean ± SEM (*n* = 5). S: saline, DW: distilled water, EtOH: ethanol, MESZS: methanol extract of Semen *Ziziphi Spinosae*, MESZS180: 180 mg/kg/d MESZS. ^###^ *p* < 0.001 vs. S/DW group; ^$$$^ *p* < 0.001 vs. EtOH/DW group (one-way ANOVA followed by Newman-Keuls post hoc test)

**Fig. 6:**
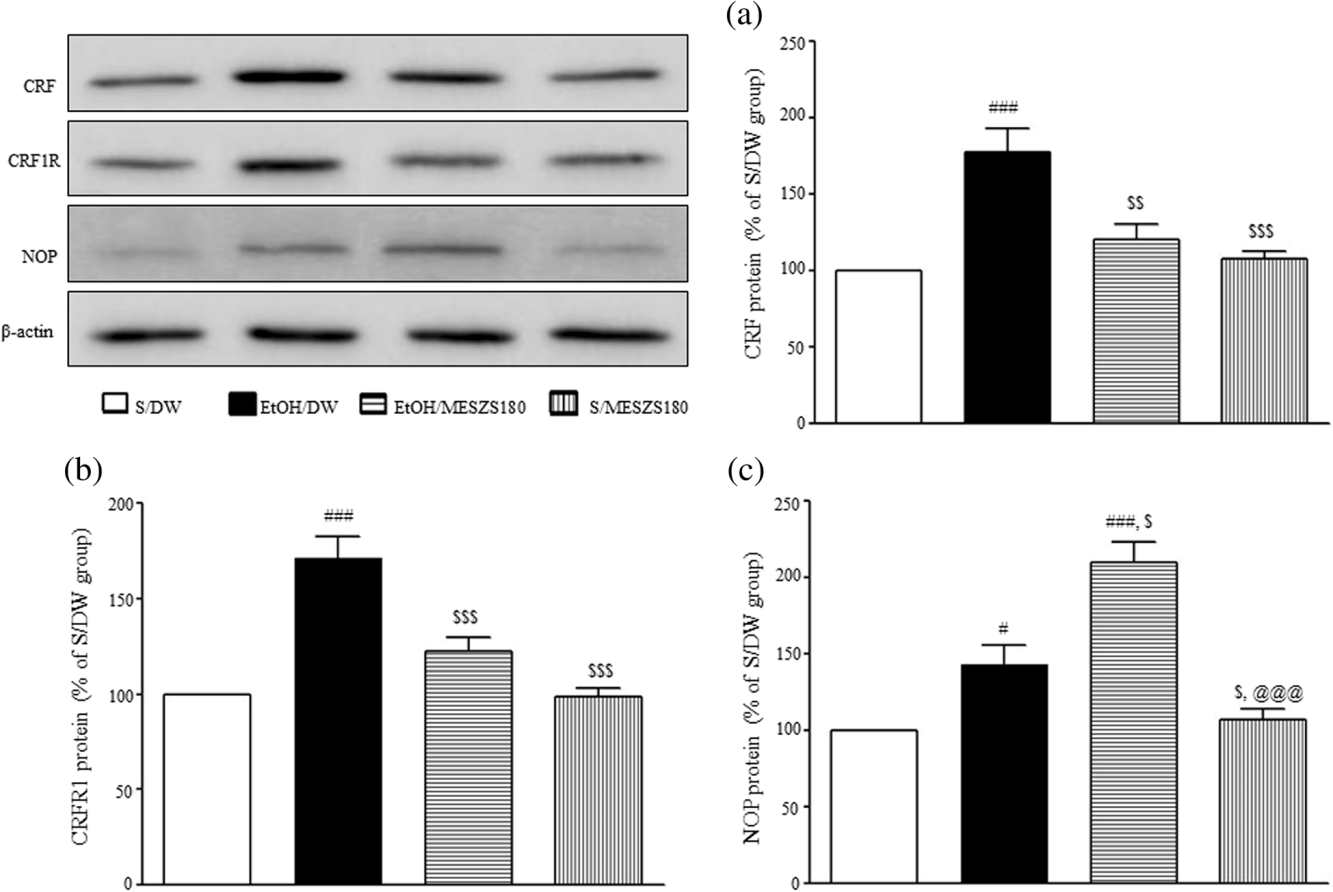
Effects of MESZS on the protein expressions of amygdaloid (**a**) CRF, (**b**) CRFR1 and (**c**) NOP during EtOHW. Immediately after the behavioral test, the protein expressions of CRF and CRFR1 and NOP in the CeA were detected using Western blot analysis. All data are expressed as a mean ± SEM (*n* = 4). S: saline, DW: distilled water, EtOH: ethanol, MESZS: methanol extract of Semen *Ziziphi Spinosae*, MESZS180: 180 mg/kg/d MESZS. ^#^ *p* < 0.05 and ^###^ *p* < 0.001 vs. S/DW group; ^$^ *p* < 0.05, ^$$^ *p* < 0.01 ^$$$^ *p* < 0.001 vs. EtOH/DW group; ^@@@^ *p* < 0.001 vs. EtOH/MESZS180 group (one-way ANOVA followed by Newman-Keuls post hoc test)

In this study, qPCR and Western blot analyses were performed to evaluate the involvement of the amygdaloid CRF/CRFR1 signaling pathway in the anxiolytic effects of MESZS during EtOHW. The qPCR analysis revealed that EtOHW significantly increased the mRNA expression levels of both CRF and CRFR1 in the CeA compared with the saline control [CRF mRNA: F_(3, 16)_ = 15.56, *p* < 0.001; saline-treated control group (100%, *n* = 5) vs. EtOH-treated control group (186.43 ± 18.41%, *n* = 5), *p* < 0.001; CRFR1 mRNA: F_(3, 16)_ = 21.32, *p* < 0.001; saline-treated control group (100%, *n* = 5) vs. EtOH-treated control group (171.14 ± 11.70%, *n* = 5), *p* < 0.001], while treatment with 180 mg/kg/d MESZS effectively inhibited these increases [CRF mRNA: EtOH-treated control group vs. EtOH/MESZS180 (115.26 ± 8.19%, *n* = 5), *p* < 0.001; CRFR1 mRNA: EtOH-treated control group vs. EtOH/MESZS180 (112.63 ± 6.83%, *n* = 5), *p* < 0.001] (Fig. 5). The Western blot analysis revealed significant increases in the CRF and CRFR1 proteins in the CeA [CRF protein: F_(3, 12)_ = 13.79, *p* < 0.001; saline-treated control group (100%, *n* = 4) vs. EtOH-treated control group (177.95 ± 15.42%, *n* = 4), *p* < 0.001; CRFR1 protein: F_(3, 12)_ = 23.20, *p* < 0.001; saline-treated control group (100%, *n* = 4) vs. EtOH-treated control group (171.56 ± 11.20%, *n* = 4), *p* < 0.001], which were paralleled by mRNA expression, but administration of 180 mg/kg/d MESZS suppressed the increases in protein expression [CRF protein: EtOH-treated control group vs. EtOH/MESZS180 (120.68 ± 9.96%, *n* = 4), *p* < 0.01; CRFR1: EtOH-treated control group vs. EtOH/MESZS180 (122.40 ± 7.25%, *n* = 4), *p* < 0.01] (Fig. 6). In addition, 180 mg/kg/d MESZS alone did not change either the mRNA expression or the protein levels of CRF and CRFR1 in the CeA (Figs. 5 and 6).

### Effects of MESZS on N/OFQ and NOP mRNA expression and NOP protein levels in the CeA

**Fig. 7:**
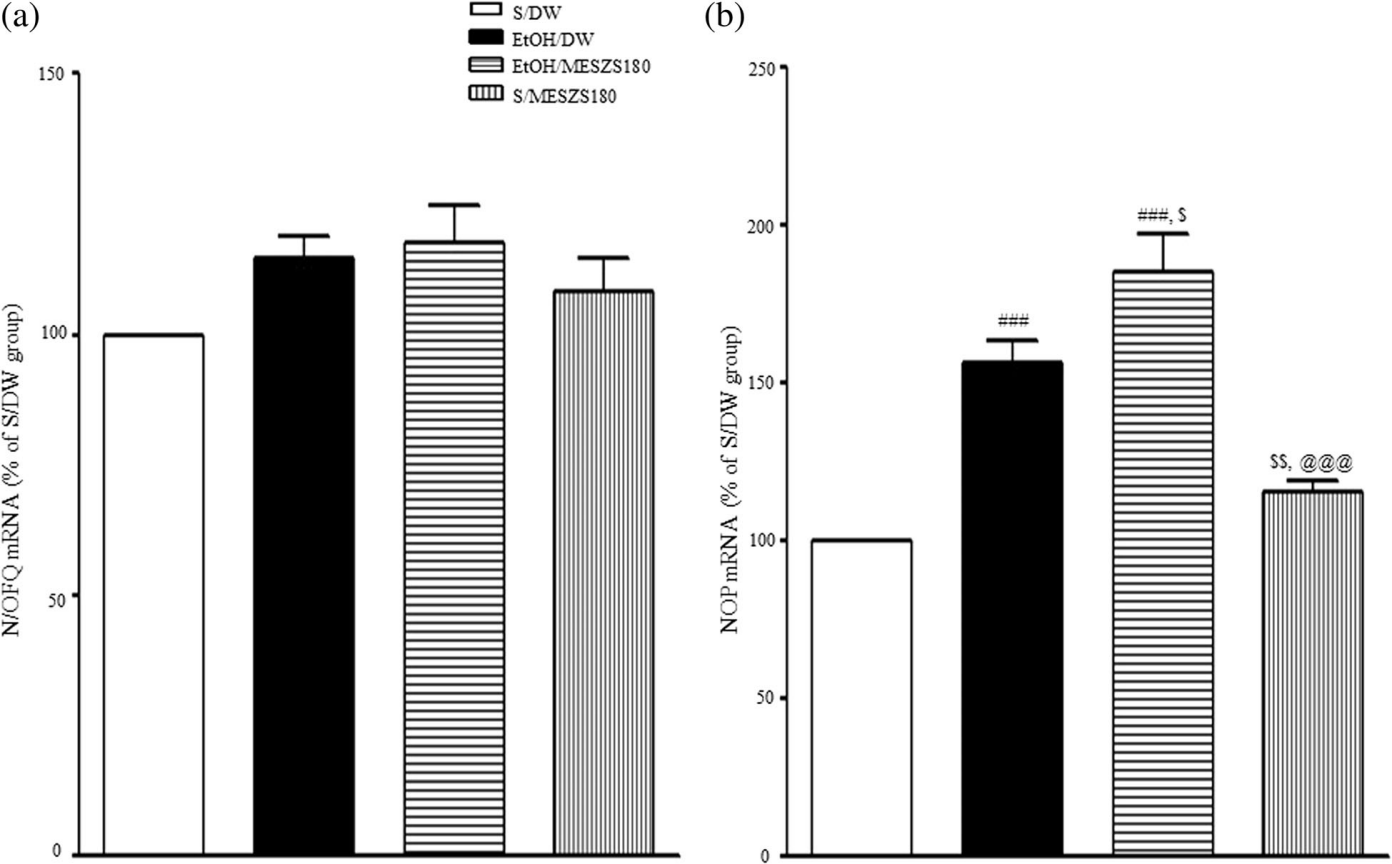
Effects of MESZS on the mRNA expressions of amygdaloid (**a**) N/OFQ and (**b**) NOP during EtOHW. Immediately after the behavioral test, the mRNA levels of N/OFQ and NOP in the CeA were evaluated using qPCR analysis. All data are expressed as a mean ± SEM (*n* = 5). S: saline, DW: distilled water, EtOH: ethanol, MESZS: methanol extract of Semen *Ziziphi Spinosae*, MESZS180: 180 mg/kg/d MESZS. ^###^ *p* < 0.001 vs. S/DW group; ^$^ *p* < 0.05, ^$$^ *p* < 0.01 vs. EtOH/DW group; ^@@@^ *p* < 0.001 vs. EtOH/MESZS180 group (one-way ANOVA followed by Newman-Keuls post hoc test)

In this study, qPCR analysis revealed no significant differences in amygdaloid N/OFQ mRNA expression levels between EtOHW rats and saline-treated control rats on the third day after terminating the EtOH treatment, and N/OFQ mRNA expression levels were also not affected by 180 mg/kg/d MESZS [N/OFQ mRNA: F_(3, 16)_ = 2.16, *p* > 0.05; saline-treated control group (100%, *n* = 5) vs. EtOH-treated control group (114.52 ± 4.27%, *n* = 5), *p* > 0.05; saline-treated control group vs. saline/MESZS180 group (108.22 ± 6.61%, *n* = 5), *p* > 0.05] (Fig. 7). However, EtOHW significantly increased amygdaloid NOP mRNA expression levels [NOP mRNA: F_(3, 16)_ = 27.26, *p* < 0.001; saline-treated control group (100%, *n* = 5) vs. EtOH-treated control group (156.16 ± 7.36%, *n* = 5), *p* < 0.001], and the 180 mg/kg/d MESZS treatment during EtOHW further promoted these increases [EtOH-treated control group vs. EtOH/MESZS180 group (185.13 ± 12.41%, *n* = 5), *p* < 0.05] (Fig. 7). Moreover, Western blot analysis confirmed that these increases in NOP gene expression levels entailed elevated NOP protein expression in the CeA, such that amygdaloid NOP protein levels were enhanced in EtOHW compared to the saline-treated controls, and the 180 mg/kg/d MESZS treatment promoted this enhancement [NOP protein: F_(3, 12)_ = 26.57, *p* < 0.001; saline-treated control group (100%, *n* = 4) vs. EtOH-treated control group (142.90 ± 12.61%, *n* = 4), *p* < 0.05; EtOH-treated control group vs. EtOH/MESZS180 group (210.30 ± 13.05%, *n* = 4), *p* < 0.05] (Fig. 6). Treatment with 180 mg/kg/d MESZS alone slightly increased both NOP mRNA and protein expression levels in the CeA, but not significantly [NOP mRNA: saline-treated control group vs. saline/MESZS180 group (115.32 ± 3.48%, *n* = 5), *p* > 0.05; NOP protein: saline-treated control group vs. saline/MESZS180 group (107.05 ± 7.35%, *n* = 4), *p* > 0.05] (Figs. 6 and 7).

### Effects of intra-CeA infusion of CRF or UFP-101 on the anxiolytic action of MESZS during EtOHW

**Fig. 8:**
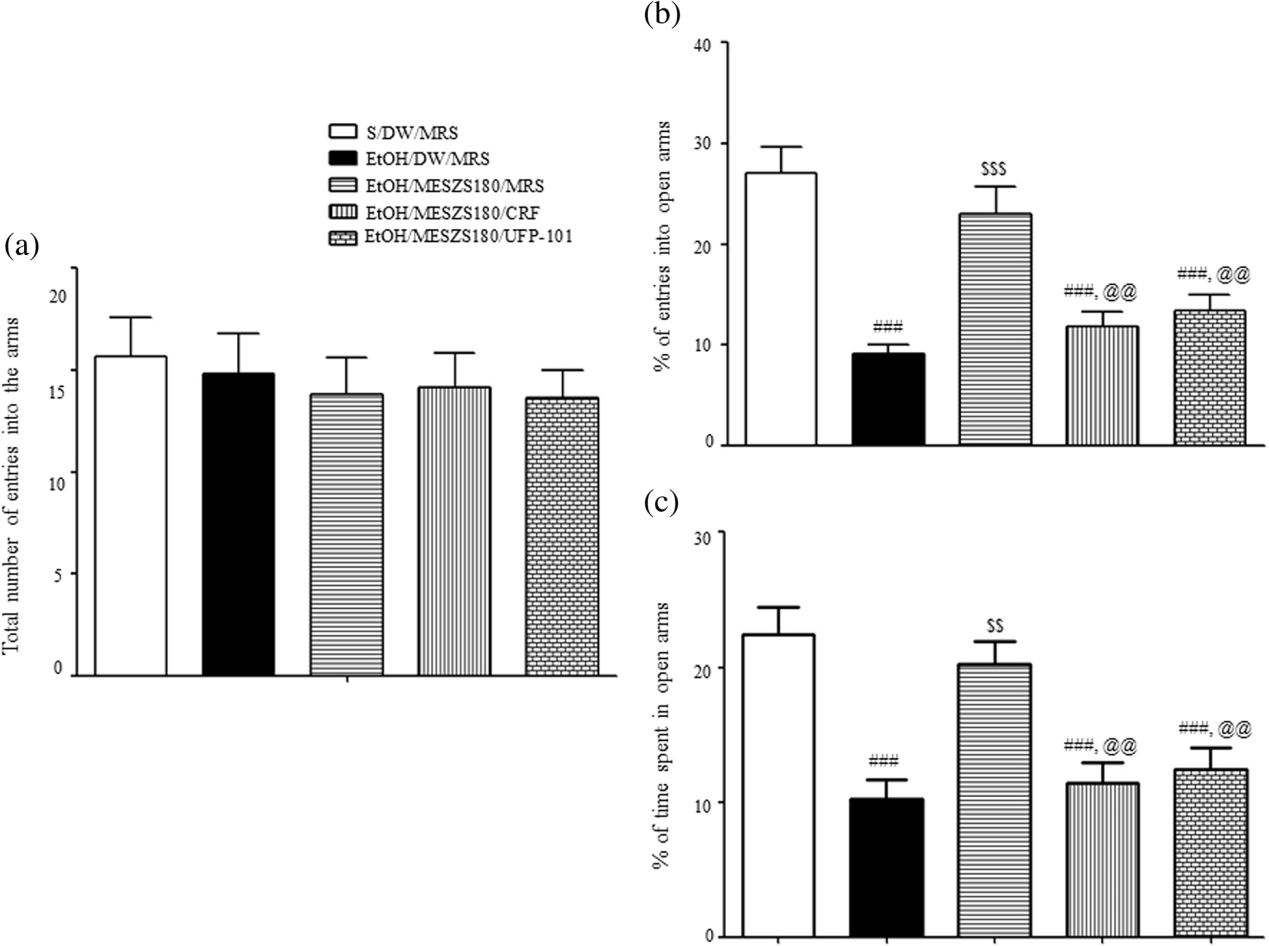
Effects of post-MESZS infusions of CRF or UFP-101 into the CeA on anxiolytic actions of MESZS in elevated plus maze (EPM) test. At 60 min after the third treatment with 180 mg/kg/d MESZS, the rats received bilateral intra-CeA infusions of CRF or UFP-101, and then were behaviorally evaluated in the EPM. (**a**) The total number of entries into (open and closed) arms of EPM by rats. (**b**) Percentage of numbers of entries into open arms. (**c**) Percentage of time spent in open arms. All data are expressed as a mean ± SEM (*n* = 6). S: saline, DW: distilled water, EtOH: ethanol, MRS: modified Ringers’ solution, MESZS: methanol extract of Semen *Ziziphi Spinosae*, MESZS180: 180 mg/kg/d MESZS. ^###^ *p* < 0.001 vs. S/DW/MRS group; ^$$^ *p* < 0.01 and ^$$$^ *p* < 0.001 vs. EtOH/DW/MRS group; ^@@^ *p* < 0.01 vs. EtOH/MESZS180/MRS group (one-way ANOVA followed by Newman-Keuls post hoc test)

Another EPM test with an additional cohort of rats bearing bilateral guide cannulae in the CeA revealed that EtOHW rats displayed anxiety-like behavior that was blocked by treatment with 180 mg/kg/d MESZS [percentage of entries into open arms: F_(4, 25)_ = 15.13, *p* < 0.001; saline/DW/MRS group (27.11 ± 2.59%, *n* = 6) vs. EtOH/DW/MRS group (9.17 ± 0.81%, *n* = 6), *p* < 0.001; EtOH/DW/MRS group vs. EtOH/MESZS180/MRS group (23.02 ± 2.73%, *n* = 6), *p* < 0.001; percentage of time spent in open arms: F_(4, 25)_ = 11.68, *p* < 0.001; saline/DW/MRS group (22.42 ± 2.00%, *n* = 6) vs. EtOH/DW/MRS group (10.26 ± 1.36%, *n* = 6), *p* < 0.001; EtOH/DW/MRS group vs. EtOH/MESZS180/MRS group (20.23 ± 1.67%, *n* = 6), *p* < 0.01], consistent with the aforementioned behavioral findings. However, the same EMP test also indicated that the anxiolytic effect of MESZS was abolished by either CRF or UFP-101 delivered into the CeA after the third dose of 180 mg/kg/d [percentage of entries into open arms: EtOH/MESZS180/MRS group vs. EtOH/MESZS180/CRF group (11.80 ± 1.48%, *n* = 6), *p* < 0.01; EtOH/MESZS180/MRS vs. EtOH/MESZS180/UFP-101 group (13.36 ± 1.68%, *n* = 6), *p* < 0.01; percentage of time spent in open arms: EtOH/MESZS180/MRS group vs. EtOH/MESZS180/CRF group (11.43 ± 1.48%, *n* = 6), *p* < 0.01; EtOH/MESZS180/MRS group vs. EtOH/MESZS180/UFP-101 group (12.46 ± 1.55%, *n* = 6), *p* < 0.01] (Fig. 8).

## Discussion

This study demonstrated that administration of 60 and 180 mg/kg/d MESZS once daily for 3 d during EtOHW dose-dependently attenuated EtOHW-induced anxiety-like behavior in rats and prevented EtOHW-induced augmentation of plasma CORT secretions. In addition, 180 mg/kg/d MESZS suppressed increases in the mRNA and protein expression levels of both CRF and CRFR1 in the CeA during EtOHW. Moreover, 180 mg/kg/d MESZS further promoted the EtOHW-induced enhancement of amygdaloid mRNA and protein expression levels of NOP. Finally, both CRF and UFP-101 locally administered into the CeA after the third dose of 180 mg/kg/d MESZS abolished the anxiolytic actions of the MESZS. Taken together, these results suggested that MESZS ameliorated EtOHW-induced anxiety in rats by improving amygdaloid CRF/CRFR1 and N/OFQ/NOP signaling.

It is well documented that crude extracts and some bioactive ingredients of SZS ameliorate basal anxiety in rodents [22, 23]. In addition, a recent study by our research team demonstrated that an aqueous extract of SZS can also mitigate nicotine withdrawal-induced anxiety [26]. In a preliminary experiment, we found that a single dose of 180 mg/kg MESZS attenuated rat basal anxiety-like behavior in the EPM test. In the present study, the OF test revealed that EtOHW rats exhibited significant decreases in the distance traveled and the time spent in the center zone compared with saline-treated control rats, while no differences were observed between the two groups in the total distance traveled by each rat, marking the occurrence of exacerbated anxiety-like behavior during EtOHW. However, both 60 and 180 mg/kg/d MESZS reversed this behavioral change in the same OF test. These results indicated that treatment with MESZS during EtOHW produced anxiolytic effects against withdrawal-induced anxiety-like behavior. The subsequent EPM test further supported these results. In the EPM test, the same EtOHW rats showed decreased numbers of entries into the open arms, along with decreased staying time in the open arms compared with their saline-treated counterparts. Similar to the OF test, both 60 and 180 mg/kg/d MESZS effectively blocked these decreases in a dose-dependent manner. Taken together, these results lead us to conclude that MESZS attenuates EtOHW-induced anxiety if administered during withdrawal.

This conclusion was also endocrinologically evaluated in this study. The CRF-adrenocorticotrophic hormone (ACTH)-CORT response to stress during EtOHW is complex. Although there is evidence showing that EtOHW time-dependently augments or blunts the CRF-ACTH-CORT response to stress [33], numerous studies have reported a sensitized CRF-ACTH-CORT response to subsequent stressors, including exposure to open areas or elevated spaces in rodents during a relative early stage of EtOHW, which is closely associated with heightened anxiety [34, 35]. In this study, the ELISA analysis revealed significantly elevated plasma CORT levels in EtOHW rats compared with saline-treated control rats, which was blocked by treatment with 60 and 180 mg/kg/d MESZS. These results corroborate the behavioral findings, suggesting that MESZS rectifies the disturbed hormonal CRF-ACTH-CORT response during EtOHW to exert anti-anxiety effects.

CRF is the initiator that activates the CRF-ACTH-CORT axis response to stress. CRF also acts on its receptors in the CeA to directly modulate local neurotransmission [36], and these two mechanisms are responsible for amygdaloid CRF mediating stress-induced affective disorders, including EtOHW-induced anxiety [37]. The CRF in the CeA originates not only from local CRF-containing neurons, but also the extra-CeA CRF-containing limbic regions, such as the paraventricular nucleus of hypothalamus and the bed nucleus of the stria terminalis (BNST) [38]. In addition, the CeA sends CRFergic projections to a variety of limbic areas, including the BNST [39]; therefore, some researchers have argued that stress-induced enhanced CRFergic activity does not entail an elevation of CRF levels in the CeA, as CeA CRF is axonally transported to the targeted areas. However, many studies have shown that noxious stimuli, such as pain stress and withdrawal from drugs of abuse, elicit an abnormal enhancement of CRFergic activities in the CeA, which contributes to stress-induced anxiety. For example, Yamano et al. [40] reported that electric foot shock stress increased amygdaloid CRF mRNA expression levels. Meanwhile, Lack et al. [41] found that chronic EtOH intake upregulated CRF mRNA expression levels in the CeA of rats. In addition, our previous study demonstrated that acupuncture treatment attenuated EtOHW-induced anxiety by inhibiting the increase in amygdaloid CRF mRNA expression [35]. In the present study, the qPCR and Western blot analyses showed that EtOHW significantly increased gene and protein expression of CRF in the CeA, while treatment with 180 mg/kg/d MESZS effectively prevented this transcriptional and translational up-regulated response. We observed previously that an aqueous extract of SZS blocked the enhancement of CRF mRNA and protein levels in the CeA [26]. In addition, Sanjoinine A promotes pentobarbital-induced sleeping by improving GABAergic transmission, which usually participates in modulation of CRF synthesis and secretion [24]. Therefore, the qPCR and Western blot results suggest that MESZS blocked EtOHW-induced upregulation of amygdaloid CRF expression, inhibiting anxiety-like behavior in EtOHW rats.

CRF produces its effects by activating two G protein-coupled receptors, CRFR1 and CRFR2, which are both distributed widely in the brain regions related to emotional stress responses. CRFR1 has a high affinity for CRF, whereas CRFR2 exhibits a low affinity to CRF but a high affinity to urocortin 2 and urocortin 3, which produce biochemically and physiologically opposite effects [42]. Evidence shows that CRF/CRFR1 signaling mainly contributes to CRF-induced activation of the CRF-ACTH-CORT axis during the stress response [43], and amygdaloid CRFR1, but not CRFR2, is responsible for stress- and EtOHW-induced anxiety-like behavior in rodents [39, 44]. The expression of CRFR1 in the CeA is vulnerable to withdrawal from drugs of abuse. The qPCR and Western blot analyses in this study showed significant increases in both mRNA and protein expression of CRFR1 in the CeA during EtOHW, consistent with previous studies [10, 26]. In addition, the same qPCR and Western blot analyses revealed that the 180 mg/kg/d MESZS treatment during EtOHW blocked the increased CRFR1 expression. These results indicate that MESZS suppresses EtOHW-stimulated upregulation of both CRFR1 gene and protein expression in the CeA, and that this action is linked with the anxiolytic effects of MESZS. Moreover, in this study, the latter was pharmacologically confirmed by the finding that post-MESZS infusion of CRF into the CeA almost completely abolished the anxiolytic effects of 180 mg/kg/d MESZS during EtOHW. These results, together with the suppressive effects of MESZS on amygdaloid CRF expression, suggest that the inhibitory effect of MESZS on CRF/CRFR1 signaling in the CeA mediates the anxiolytic effect of MESZS during EtOHW.

Under the normal physiological response, the brain homeostatically mobilizes anti-CRF (anti-stress) mechanisms such as N/OFQ and neuropeptide Y to curb EtOHW-induced stress response, while the imbalance tilted toward pro-stress seems to underlie EtOHW-induced anxiety. Although opposing evidence exists, robust evidence shows that agonizing central N/OFQ/NOP signaling, particularly activating the NOP receptors in the extended amygdala such the CeA and BNST, produces anti-stress and anxiolytic effects [45–47]. Unlike the NPY system that usually exhibits a decreased activity in the CeA during EtOHW [48], evidence reveals that the NOP transcripts in the CeA are increased in response to both acute restraint stress and EtOHW, and that intra-CeA infusions of N/OFQ reduce stress- and EtOHW-induced anxiety and excessive alcohol drinking in rats via amygdaloid mechanisms [49–51]. These reports collectively propose that the activity of amygdaloid N/OFQ system is increased alongside with the CRF system during EtOHW, but not enough to thwart the CRF-oriented stress response. In agreement with this proposal, the qPCR and Western blot analyses in the present study revealed significant increases in both mRNA and protein expression of NOP in the CeA during EtOHW, and treatment with 180 mg/kg/d MESZS further promoted these increases. Evidence shows that the N/OFQ/NOP system modulates the neuroendocrine stress response in a phasic, not tonic, manner [52]; therefore, it is reasonable that EtOHW increases NOP expression in the CeA because the N/OFQ/NOP system must be functionally recruited to counteract the excessive activation of the amygdaloid CRF system during EtOHW. As such, exogenously agonizing or intensifying N/OFQ/NOP signaling antagonizes CRF/CRFR1 action to produce an anti-anxiety effect. This notion was further supported in this study by the result that post-MESZS injection of UFP-101 into the CeA abrogated the anxiolytic effect of 180 mg/kg/d MESZS during EtOHW. In the present study, neither EtOHW nor treatment with 180 mg/kg/d MESZS significantly affected N/OFQ mRNA expression in the CeA. This is in agreement with the report by Auja et al. [50] that there is no significant difference in amygdaloid N/OFQ mRNA expression between EtOH-dependent rats and nondependent rats 7 d after terminating EtOH, indicating that amygdaloid N/OFQ is less vulnerable to EtOHW than NOP receptors. Taken together, these findings suggest that recruitment of the N/OFQ/NOP system during EtOHW, and the 180 mg/kg/d MESZS treatment further promotes this functional engagement to produce the anxiolytic effect.

It is worth mentioning that as seen in the HPLC profile, the MESZS used in the present study contains spinosin, magnoflorine, jujuboside A, and 6′′′-feruloyl spinosin, the same compounds included in an aqueous extract of SZS that was anxiolytic during nicotine withdrawal in a previous study [26]. In addition, there is evidence that some of these constituent compounds have therapeutic effects against spontaneous anxiety and stress-induced behavioral disorders. For example, spinosin and magnoflorine and jujuboside A can mitigate anxiety-like behavior in naive mice [53–55], and spinosin and jujuboside A improve Aβ_1–42_-induced memory loss in mice [56, 57]. These facts collectively imply that the anxiolytic effect of the MESZS involves these compounds, however, which were not individually examined in the present study. Therefore, it highlights the need for further research to identify and confirm the actual therapeutic agents in the MESZS to facilitate the discovery and development of new phytomedicines to treat EtOH-induced anxiety.

## Conclusions

This study demonstrated that treatment with MESZS at doses of 60 and 180 mg/kg/d dose-dependently attenuated EtOHW-induced anxiety and inhibited the excessive secretion of plasma CORT during EtOHW. Moreover, 180 mg/kg/d MESZS prevented EtOHW-induced increases in the protein and mRNA expression of CRF and CRFR1 and further augmented EtOHW-induced increases in NOP mRNA expression and protein levels in the CeA. These results suggest that SZS blocks EtOHW-induced anxiety by improving CRF/CRFR1 and N/OFQ/NOP transmission in the CeA, therefore, can be considered to be a promising candidate for developing new drugs to treat alcoholism.

## Additional file

Additional file 1: Positions of CeA cannulae. (TIF 4223 kb)

## Data Availability

The data supporting the conclusions in this study are statistically analyzed and presented in Results section, and the related raw data are also available from the corresponding author on reasonable request. All materials used in this study are properly included in Methods section.

## Abbreviations

CeA: Central nucleus of amygdala
CORT: Corticosterone
CRF: Corticotropin-releasing factor
CRFR1: Corticotropin-releasing factor receptor 1
DW: Distilled water
EPM: Elevated plus maze
EtOH: Ethanol
EtOHW: Ethanol withdrawal
MESZS: Methanol extract of Semen *Ziziphi Spinosae*
MESZS60 (180): 60 (180) mg/kg/d MESZS
MRS: Modified Ringer’s solution
N/OFQ: Nociceptin/orphanin FQ
NOP: Nociceptin/orphanin FQ receptor
OF: Open field
ppN/OFQ: prepro-N/OFQ
SZS: Semen *Ziziphi Spinosae*
UFP-101: [Nphe^1^, Arg^14^, Lys^15^] nociceptin-NH_2_
