# Supplementary figures and images for: Methanol extract of semen Ziziphi Spinosae attenuates ethanol withdrawal anxiety by improving neuropeptide signaling in the central amygdala
Source: BMC Complement Altern Med. 2019 Jun 24;19:147. doi: 10.1186/s12906-019-2546-0 (PMC6591875; doi:10.1186/s12906-019-2546-0)

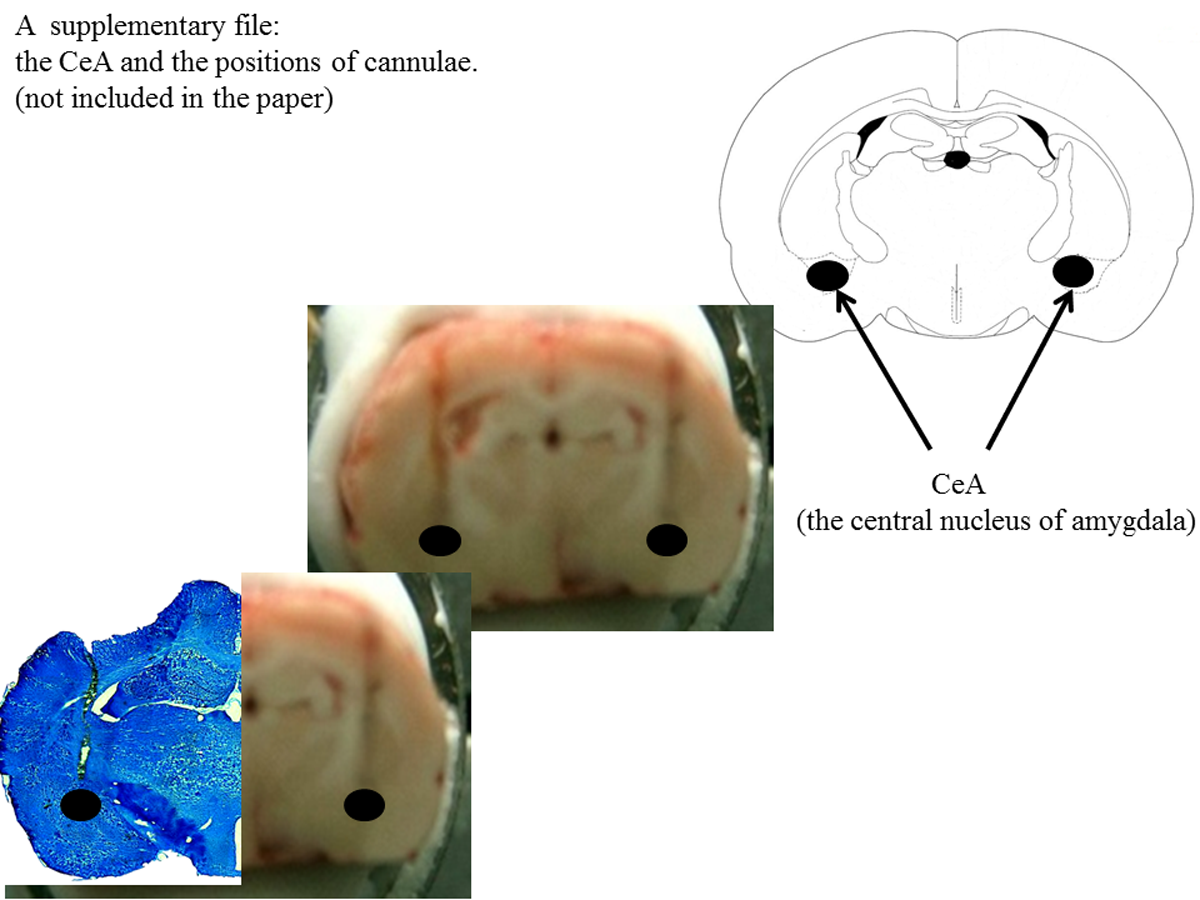

Supplement: Supplementary file 1 — Positions of CeA cannulae. (TIF 4223 kb) [file 12906_2019_2546_MOESM1_ESM.tif]
